# Behavioral and physiological indicators of stress in horses during an equine-assisted learning program for youth with a history of trauma

**DOI:** 10.1093/tas/txaf027

**Published:** 2025-02-27

**Authors:** Sarah K Matlock, Ashley Singh, Temple Grandin, Tamara Merritt, Terry Nett, Sarah Jean Reega, B Caitlin Peters

**Affiliations:** Department of Animal Sciences, Colorado State University, Fort Collins, CO 80523, USA; Department of Animal Sciences, Colorado State University, Fort Collins, CO 80523, USA; Department of Animal Sciences, Colorado State University, Fort Collins, CO 80523, USA; Hearts & Horses Therapeutic Riding Center, Loveland, CO 80537, USA; Department of Biomedical Sciences, Colorado State University, Fort Collins, CO 80523, USA; Department of Animal Sciences, Colorado State University, Fort Collins, CO 80523, USA; Department of Animal Sciences, Colorado State University, Fort Collins, CO 80523, USA; Department of Occupational Therapy, Colorado State University, Fort Collins, CO 80523, USA

**Keywords:** equine-assisted services, equine-assisted learning, stress, welfare, horse

## Abstract

Equine-assisted learning (EAL) is gaining in popularity due to its demonstrated benefits to participants, including increased social-emotional competencies and self-efficacy, and decreased symptoms of depression. Accordingly, EAL is increasingly utilized as a method to build soft skills for people who have a history of trauma and/or who may struggle with emotional regulation. Within the context of equine-assisted services (EAS) broadly, there is some evidence that participants who have trauma and/or emotional dysregulation may cause increased stress to horses when compared to interactions with other types of EAS participants (e.g., participants with cognitive delays, physical disabilities, etc.). It is important to understand the impacts of EAL for individuals with a history of trauma on the well-being of the horse. The purpose of this pilot study was to identify and compare the presence of behavioral and physiological indicators of stress in horses integrated into an EAL program for youth with a history of trauma who struggle with emotional regulation (n = 11) compared to the control condition of an EAL program for young adults with developmental delays (n = 7). Indicators of stress included salivary cortisol, eye temperature, and equine behaviors indicative of stress. We did not find significant differences in the indicators of stress in the horses when interacting with the youth with a history of trauma compared to the control condition (salivary cortisol, p = 0.55; eye temperature, p = 0.39; behavioral indicators of stress, p = 0.81). Contrary to previous findings, we did not find evidence that EAL with youth with a history of trauma increased the stress of the participating horses in comparison to EAL for a different population (young adults with developmental disabilities). Furthermore, we also found that physiological measures of stress were within normal ranges for both the experimental condition and control condition, providing further evidence that EAL does not appear to increase physiological stress in horses beyond normal ranges.

## Teaser

This research demonstrates that interacting with youth with a history of trauma and emotional dysregulation did not increase stress in horses during an equine-assisted learning program, compared to a control condition of equine-assisted learning for individuals without a history of trauma.

## INTRODUCTION

The integration of horses into human health has a long and rich history that predates the 20th century. The unique benefits of the horse’s movement were even described by Hippocrates in his book Natural Exercise as early as 460 BC (Adams, 1886). Moreover, the gain in popularity over the past several decades has led to a significant increase in scholarly work, resulting in more systematic ways to oversee services (e.g., providing certification pathways for increasing credibility (Professional Association of Therapeutic Horsemanship International, 2023)), and clearly define services (Wood et al., 2021). These scientific advancements have also led to the development of a universal term, equine-assisted services (EAS), referring to all types of human-equine interactions where professionals integrate horses to benefit people (Wood et al., 2021). Although this dramatic growth in scholarly work supporting the benefits of EAS on human health is exciting, the welfare of EAS horses compels equal attention. Problematic behavioral issues potentially indicative of service burnout in EAS horses are common reasons for EAS horse retirement (Rankins et al., 2021), driving the need to better understand stressors specific to this population of horses.

Promisingly, a growing body of research has begun to examine the welfare of EAS horses, with preliminary evidence indicating that horses participating in EAS do not exhibit increased physiological or behavioral markers of stress, neither in general, nor when compared to other equine activities such as recreational riding (Kaiser et al., 2006; Nobbe, 2016; Ayala et al., 2021). These consistent scholarly findings provide increasing confidence that EAS, by and large, are not inherently stressful for horses. Yet, problem behaviors during services persist; problematic behaviors such as biting, pinning ears, or kicking are cited as the most frequent reason for retiring EAS horses (Rankins et al., 2021). A closer look at the research reveals that the type of participant (rather than the type of service) might be implicated in increased negative stress for EAS horses. For example, in one study horses integrated into services with participants with a history of trauma experienced an increase in heart rate (Merkies et al., 2018). Further, Kaiser et al. (2006) found that horses demonstrated increased behavioral indicators of stress during EAS for a population they characterized as “at-risk children” in comparison to all other EAS populations. Indeed, this mirrors previous findings in equitation sciences implicating human emotions’ effect on equine behavior, whereby humans expressing negative emotions can cause stress to horses, and can even lead to horse-related accidents due to fear (Trösch et al., 2019). Previous research has considered equine stress while interacting with individuals with a history of trauma or “at-risk children” in a round pen (Merkies et al., 2018) or during various mounted EAS (Kaiser et al., 2006), but there has been no investigation into equine stress during interactions at the tie rail compared to during arena work, an important consideration given that many EAS include grooming and tacking prior to riding. One study has investigated equine behavioral and physiological reactions to “at-risk adolescents” during EAS over the course of several weeks, finding that horse heart rate increased over the course of the 10-wk study period, while affiliative behaviors decreased from Week 1 to Week 10 (Arrazola and Merkies, 2020). Thus, the impact of time is a relevant consideration given that many EAS programs last for several weeks/months and equine stress reactions to individuals with a history of trauma and/or emotional dysregulation may change with increased exposure to the participant over weeks/months.

One specific type of EAS, equine-assisted learning (EAL), integrates horses into learning programs to help learners achieve specific educational objectives (Wood et al., 2021). Equine-assisted social-emotional learning is increasingly utilized as a method to build social-emotional skills for people who have a history of trauma and/or who may struggle with emotional regulation; demonstrated benefits to participants include improved social-emotional competencies and self-efficacy, and decreased symptoms of depression (Frederick et al., 2015; Ho et al., 2017; Perkins, 2018). Social-emotional learning programs and/or psychotherapy services which integrate horses often include participants with histories of trauma due to the positive benefits these programs have on mental health of participants. However, research in human trauma provides robust support for the connection between traumatic exposure and emotional dysregulation (Berfield et al., 2022). Preliminary findings indicate that EAS for people with a history of trauma and/or emotional dysregulation may negatively impact equine welfare, observed through behavioral and physiological indicators of stress in horses (Kaiser et al., 2006; Merkies et al., 2018). For these reasons, it is important to further investigate this connection between human trauma and equine welfare within the specific context of EAS.

The purpose of this study, therefore, was to evaluate behavioral and physiological indicators of stress in horses integrated into a social-emotional learning program for youth with a history of trauma. Based on previous findings, we expected to see increased indicators of stress in horses integrated into this EAL program due to the potential effects of the participant’s emotional dysregulation on the stress-levels of the horse. To explore this relationship further, we asked the following questions:

1. Do horses demonstrate increased stress indicators during EAL for youth with a history of trauma compared to during an EAL control condition for individuals without a history trauma?
2. Do horses demonstrate increased stress during three specific phases of EAL for youth with a history of trauma (baseline, tie rail, arena work) compared to during the same phases of an EAL control condition?
3. Does equine stress change across time of an 8-wk EAL program for youth with a history of trauma compared to across time of an 8-wk EAL control condition?

## MATERIALS AND METHODS

Colorado State University’s IRB approved all study procedures regarding human data collection (#3473). Colorado State University’s Institutional Animal Care and Use Committee exempted this study from full board review due to the unimpactful and noninvasive observation and data collection methods. The management of the horses in this study adhered to the Professional Association of Therapeutic Horsemanship International (PATH, Intl.) guidelines for the ethical treatment and care of horses integrated into human services. PATH Intl. is the leading agency for supporting the advancement of professional equine-assisted services through evidence-based standards, credentialing and education (Professional Association of Therapeutic Horsemanship International, 2023).

### Program Description

This study was conducted at Hearts & Horses, Inc., a PATH, Intl. Premier Accredited Center that provides a variety of equine-assisted services. Hearts & Horses has partnered with a local school district to provide a social-emotional learning program, known as *Changing Leads*, for youth who have been previously identified as having a history of trauma and are not responding to efforts for improving social-emotional competencies within the school setting. While previous research may have used the term youth “at-risk” to describe this population, we have elected not to use that term due to scholarly criticism that the term further marginalizes under-served populations and is not well-defined (Schonert-Reichl, 2000; Riele, 2006; Pica-Smith and Veloria, 2012). *Changing Leads* is an 8-wk social and emotional learning program designed to help youth who struggle with emotional regulation by building soft skills (e.g., self-awareness, social-awareness, and personal responsibility) through a partnership with the horse. Prior to the beginning of the program, there is a meet-and-greet session at participants’ schools so staff can learn more about the student. The program is then guided by Certified Therapeutic Riding Instructors (CTRIs) dually certified as Equine Specialists in Mental Health and Learning (Professional Association of Therapeutic Horsemanship International, 2023). The program occurs once a week for 8 consecutive weeks for 3.5 h, including classroom time, lunch, and 1.5 h of EAL. Students begin the morning in a classroom-based group activity and then are led to the barn for 1.5 h of EAL. Within this activity, students are divided into smaller groups of 3 to 4 participants, with each group consisting of both a lead and support CTRI, as well as one adult volunteer horse leader per student. CTRIs put great intention into pairing each student with a horse and adult volunteer that best suits their needs, and these partnerships are maintained for the eight-week program. The EAL activities are centered on the specific social and emotional competency of the week, and include: 1) a warm-up, 2) a description and demonstration of the activity, 3) practicing the activity, 4) a cool-down centered on reflection. The EAL activities are both mounted and unmounted.

### Site Description and Equine Management

Table 1 provides equine characteristics for horses included in this study. The horse management team at Hearts & Horses adhered to optimal practices in equine management, meeting and exceeding PATH Intl. guidelines. The research team assessed these conditions under the standards of the Five Domains of Animal Welfare (Mellor et al., 2020) to ascertain potential environmental and/or management circumstances that might impact behavior during sessions. Hearts & Horses’ herd were fed forage 4 times per day with the evening feeding in a feedbag to last longer overnight. Although the horses were individually stalled, which could contribute to confinement-related stress, they were provided with approximately 6 h of conspecific turnout each day in a larger dry lot. Many of these horses were second career and/or were older (18+ yr) and have resulting arthritis and/or other conditions associated with prior activities. Therefore, 4 of the 11 horses were provided with routine pain-relief medications, such as non-steroidal anti-inflammatories, or ulcer treatment or prevention plans (Table 1). All horses were on a 6 to 8-wk farrier cycle, up to date on veterinary care, received routine acupuncture, massage and chiropractic care when needed, and each horse had been professionally fitted to saddles. Hearts & Horses provided handling training to their CTRIs and horse leader volunteers, which included non-aversive approaches in handling. The CTRI team tracked chronic signs of mental stress (e.g., bite threats, failure to respond to cues, etc.) during services and temporarily removed any horse exhibiting these concerns. Furthermore, Hearts & Horses limits all horses to a maximum of 8 h/wk of work, far below the PATH Intl limit of 6 h/d (Professional Association of Therapeutic Horsemanship International, 2021), and provides regular days off.

**Table 1.** Equine characteristics

Name	Sex	Age, yrs	Breed	Years in EAS	Medications that could affect cortisol
Rocket	Gelding	25	Quarter Horse	4	Daily Previcox
Nugget	Gelding	9	Quarter Horse	1	N/A
Varsity	Gelding	10	Gypsy Vanner	7	N/A
Niko	Gelding	15	Quarter Horse	<1 yr	N/A
Mohica	Mare	17	Quarter Horse	2	N/A
Wrangler	Gelding	20	Paint	<1 yr	Daily Previcox
Ben	Gelding	21	Connemara	6	N/A
Apollo	Gelding	18	Percheron	<1 yr	Daily Previcox
Hope	Mare	6	Haflinger	2	N/A
Leroy	Gelding	15	Warmblood	<1 yr	Daily Previcox
Ray	Gelding	21	Quarter Horse	<1 yr	N/A

### Design

This study implemented a single-sequence 6-period (A-B-A-B-A-B) crossover design to evaluate behavioral and physiological indicators of equine stress during two different conditions. Accordingly, the same horses were repeatedly integrated into two different EAL conditions: Condition A) a social-emotional learning program for youth with a history of trauma and persistent social-emotional needs (EAL Social-emotional), and Condition B) a similar EAL program for participants without a history of trauma (EAL Control). Thus, the same horses were exposed to both conditions: the EAL Social-emotional condition (Condition A; n = 11) and the EAL Control condition (Condition B; n = 7). We collected data during three days for each condition, thus the A-B-A-B-A-B design.

The EAL Social-emotional condition consisted of eleven youth, ages 12 to 15, with a history of mild to severe trauma and who were not responding to social-emotional programming within the school. This group was also identified by counselors as students who struggle with emotional regulation, frequently miss school, and suffer from self-reported anxiety and/or depression. Table 2 provides characteristics and demographics for the youth who participated in EAL Social-emotional condition. Participants were paired with the same horse throughout the duration of the study.

**Table 2.** EAL participant demographics

Participant Characteristic	Youth in EAL Social-emotional (N = 11)	Youth in EAL Control (N = 7)
Gender: m/f/non-binary/trans	4/5/1/1	2/5/0/0
Age: mean (range)	13 (12 to 14)	20 (19 to 21)
Race: White/not reported	10/1	5/2
Hispanic or Latino: yes/no	3/8	4/3
Previous Riding Experience: yes/no/not reported	4/6/1	6/1/0
On current Medications: yes/no/not reported	6/4/1	6/1/0
Physical Disabilities: yes/no/not reported	0/9/2	2/5/0
Diagnosis:		
ADHD	5	
Autism Spectrum Disorder	2	2
Anxiety	5	2
Depression	2	
Dyslexia	1	
Developmental Delays		1
Down Syndrome		1
Intellectual Disability		2
Mood/ Personality Disorder	2	
PTSD	1	
Smith Magenis Syndrome		1
Household size: median (range)	5 (2 to 6)	3 (3 to 6)
History of: (yes/no/not reported)		
Parent-reported Trauma	3/8/0	0/6/1
Behavioral Concerns	6/5/0	2/4/1
Emotional/Mental Health Concerns	9/2/0	1/6/1
TESI Score: mean (range)	6.3 (1 to 11)	N/A

TESI = Traumatic Events Screening Inventory.

The EAL Control condition was a convenience control condition consisting of an EAL program for young adults with developmental disabilities. This population has a low incidence of emotional dysregulation, trauma and/or psychological diagnoses compared to the EAL Social-emotional condition. Hence, the EAL Control condition represents participants who traditionally participate in EAL, but do not have a history of trauma or social-emotional concerns. Further, individuals in the EAL Control condition did not have any reported physical disabilities. Similarly to the experimental condition, the EAL Control condition occurred for 1.5 h once a week for 8 wk on a different day than the experimental condition. Table 2 provides characteristics and demographics for the young adults who participated in the EAL Control condition. Participants were paired with the same horse throughout the duration of the study. There were only 7 EAL control participants, which explains why only 7 horses were exposed to this condition.

### Data Collection

**Figure 1.**
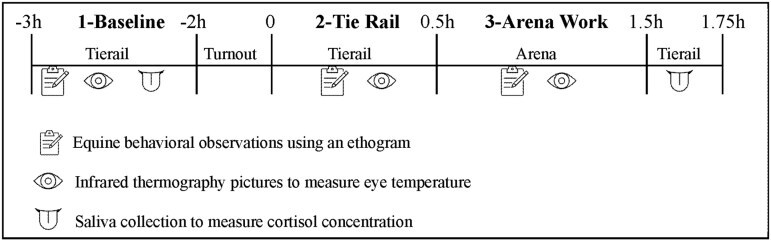
Data collection timeline. Researchers recorded equine behaviors, captured infrared thermography pictures, and collected saliva during Phase 1 at the tie rail 2 to 3 h before the EAL program began. Horses were then turned out to pasture until the EAL program began. Researchers recorded equine behaviors and captured infrared thermography pictures during Phase 2 at the tie rail with EAL participants and during Phase 3 in the arena with EAL participants. After the EAL session ended, researchers collected saliva to measure salivary cortisol concentrations.

One month prior to data collection, we piloted saliva collection with the horses in the study. During this pilot, all horses allowed the investigators to insert the swab into the side of their mouth without displaying any signs of stress. One week prior to data collection, we piloted behavioral coding and IRT capture with the seven horses in the EAL Control condition; the 4 horses only exposed to the EAL Social emotional condition were not present during this pilot due to scheduling. During the 8-wk EAL Social-emotional and EAL Control programs, we collected data on weeks three, five, and seven. This allowed time for the horses to accommodate to the program routines in weeks one and two so as not to capture possible stress due to novelty of participating in the program. Furthermore, weeks three, five and seven were all similar in structure, including both unmounted and mounted activities. Data were collected from the horses in an indoor arena at three timepoints: 1) Baseline (1-B) at the tie rail 2 to 3 h before interacting with EAL participants, 2) Tie Rail (2-TR) at the tie rail while EAL participants groomed and tacked the horses, and 3) Arena Work (3-AW) in the arena with EAL participants, which included both mounted and unmounted activities. Figure 1 provides a diagram of when data were collected. Prior to 1-B Baseline, horses were in their home box stall, where they are typically fed each morning. In between 1-B Baseline and 2-TR Tie Rail, horses were turned out to pasture, in accordance with their typical morning routine.

#### Infrared Thermography (IRT).

SR took pictures of the right eye of each horse using a FLIR E6-XT Handheld IRT camera during each of the three phases. This camera features 160 × 120 thermal resolution and has a temperature range of −20 to 550 °C temperature range. To ensure proper use of the IRT camera, SR was trained by SM, who has obtained Infrared Thermography Certification (Infrared Training Center, 2022). All photos were captured at a 90-degree angle approximately one meter away from the medial canthus of the horse’s eye. In Phase 1-B, a photo was captured from each horse alone in the tie rail, before any interaction with EAL participants. In Phase 2-TR, SR captured a photo while the horses were being groomed in the tie rail by an EAL participant, approximately 5 min after grooming began. Lastly in phase 3-AW, SR captured a photo of each horse in the arena approximately 10 min after the youths had mounted the horse. A research assistant recorded the current temperature and humidity using a thermo-hygrometer in the same working area as the horses while each photo was captured. The data was then analyzed in FLIR Thermal Studio Suite (Teledyne FLIR, 2024), a thermal imaging analysis and reporting software.

#### Salivary cortisol.

Saliva samples were collected from each horse using the SalivaBio Children’s Swab, validated for use with salivary cortisol (Salimetrics, State College, PA, Item No 5001.06). Saliva was collected from each horse in the morning around 9:00 a.m. at the tie rail, at least one-hour after the horses had their breakfast and before EAL participants interacted with the horse (1-B). Saliva samples were also collected immediately at the end of the session day at the tie rail at approximately 1:00 p.m. To collect saliva, the swab was inserted into the side of the horse’s mouth and was held in the cheek for 35 s. Samples were immediately stored on ice in a cooler, and then transported and stored in a freezer at −20-degrees Celsius until they were shipped to Salimetrics overnight on dry ice for analysis. Samples were assayed at the Salimetrics’ SalivaLab (Carlsbad, CA) using the Salimetrics Salivary Cortisol Assay Kit (Cat. No. 1-3002), a high sensitivity enzyme immunoassay. Samples were thawed to room temperature, vortexed, and then centrifuged for 15 min at approximately 3,000 RPM (1,500 x g) immediately before the assay was performed. The assay had a lower limit of sensitivity of 0.007 μg/dL, a standard curve range from 0.012 to 3.0 μg/dL, an average intra-assay coefficient of variation of 4.60%, and an average inter-assay coefficient of variation 6.00%.

#### Behavior ethogram.

We used an applied ethogram to capture behavioral indicators of low-mid level tension or stress in horses. The applied ethogram included the 15 equine behaviors defined in Table 3, drawn from previous literature (Seaman et al., 2002; McDonnell, 2003; Draaisma, 2017; Torcivia and McDonnell, 2021). Two researchers coded equine behaviors simultaneously during all three phases. Researchers employed a scan sampling technique (Martin and Bateson, 1986), during which researchers observed a single horse for one minute, then observed a subsequent horse for one minute, until all horses were observed. The number of intervals that researchers observed each horse varied by phase, given that each phase lasted a different length of time (Figure 1). In Phase 1-B each horse was observed for two 1-min intervals after saliva collection occurred; in Phase 2-TR each horse was observed for a one 1-min interval during grooming or tacking; and in Phase 3-AW, each horse was observed for three 1-min intervals during mounted and unmounted activities. Phase 3 observations never occurred during mounting. Researchers recorded each behavior as either absent or present during each observation interval (i.e., one-zero sampling, Martin and Bateson, 1986). SM and AS established interrater reliability on use of the applied ethogram by obtaining 94% agreement on observed behaviors. In instances of disagreements between the two raters, researchers discussed the discrepancies and came to a joint consensus.

**Table 3.** Applied ethogram of behaviors indicative of stress during equine-assisted services

Behavior	Definition
Licking and Chewing	Cluster of autonomic responses in the mouth following an acute sympathetic surge, including salivation (leading to chewing movements, swallowing, tongue extensions) (Torcivia and McDonnell, 2021).
Jaw and/or Body Stretch	Opened mouth with or without uncovering the teeth nor protruding tongue (Draaisma, 2017). Ridged extension of the limbs, arching of the neck and back (McDonnell, 2003).
Neck and/ or Body Shake	Rapid, rhythmic rotation of the whole body or just the head and neck along the long axis (Torcivia and McDonnell, 2021).
Lowering the Head and Neck	Horse rapidly (< 5 s) moves its head and neck into a position that places the neck in a lowered position, from any starting point
Itching (Rubbing) Head and Neck	Rubbing face against forelimb (autogrooming) (Torcivia and McDonnell, 2021).
Head Raise	Horse rapidly (< 5 s) moves its head and neck into a position that places the neck in a raised position, from any starting point
Pawing	Reaching a forelimb cranially and dragging the hoof along or above the substrate while sweeping caudally, often in rhythmic series (Torcivia and McDonnell, 2021).
Step Forward/ Back	One step forward or one step back with either front or hind limb while stationary (McDonnell, 2003).
Ears Pinned	Ears pressed caudally against the head and neck (McDonnell, 2003).
Head Turn with Ears Pinned	Head turned horizontally to handler or object, with ears pinned; exaggerated turn that deviates from relaxed state.
Tail Swish	Moving tail suddenly from side to side (Torcivia and McDonnell, 2021).
Head Toss	Quick rotational toss of the head (Torcivia and McDonnell, 2021).
Kick Threat	Intent to kick, swinging rump, backing up, or stamping hind leg toward stimulus (Seaman et al., 2002).
Bite Threat	Bite intention movement of the head and mouth with ears back and neck extended, with no actual contact (Seaman et al., 2002).
Lip Quiver	Involuntary movement (twitching) of the lips, often with relaxation (drooping) of the lower lip (Torcivia and McDonnell, 2021).

For analyses, we summed all 15 behaviors into one total score of the number of unique stress behaviors during the 1-min observation interval. We then averaged the total scores across the observation intervals in each phase (two intervals in Phase 1 and three intervals in Phase 3), resulting in a final response variable indicating the average number of unique stress behaviors per minute in each phase.

#### Human participant characteristics.

We collected the following demographic information from EAL participants using the Hearts & Horses enrollment form: age; race; ethnicity; gender; medications; family household size; medical diagnoses; and history of trauma, emotional or behavioral concerns. Furthermore, caregivers of youth in the EAL Social-emotional condition completed the Traumatic Events Screening Inventory—Parent Report Revised, a 28-item parent-report checklist that assesses a child’s exposure to potentially traumatic events in three categories: 1) accident, disaster, or illness, 2) physical maltreatment, and 3) sexual maltreatment. This tool has been validated as a parent-report measure of adverse childhood experiences (Choi et al., 2019). Parents are asked to report yes, no, or unsure about their child’s exposure to a variety of potentially traumatic events, and “yes” answers are scored as 1; a score of 4 or higher indicates significant risk to mental, emotional and/or social functioning (Roberts et al., 2013). Some, but not all, participants in each condition had previous experience with horses (Table 2).

### Data Analysis

Analyses were completed using SAS 9.4 (SAS Institute Inc., Cary NC, 2023). Summary statistics were calculated for each variable, condition, week, and phase. A mixed model was fit for each response variable separately. Specifically, fixed effects included condition (EAL Social-emotional or EAL Control), week (3, 5 or 7), phase (1-B, 2-TR or 3-AW), and all two-way interactions. Horse was included as a random effect to account for repeat observations of horses. F-tests for fixed effects were used to determine pairwise comparisons of interest. Tukey adjusted p-values were used when comparing factors with more than two levels, with *p* < 0.05 the threshold for significance. Residual diagnostic plots were used to confirm model assumptions of normality and equal variance.

To answer research question 1, we examined main effects of condition to determine if equine stress differed during EAL Social-emotional compared to EAL Control. To answer research question 2, we examined condition × phase interaction effects to determine if equine stress differed during different phases (1-B, 2-TR, or 3-AW) of EAL Social-emotional compared to EAL Control. To answer research question 3, we examined condition by week interaction effects to examine if equine stress during the EAL Social-emotional condition differed over the course of 5 wk in comparison to the EAL Control condition.

## Results

### Research Question 1: Equine Stress During EAL Social-Emotional vs EAL Control

**Figure 2.**
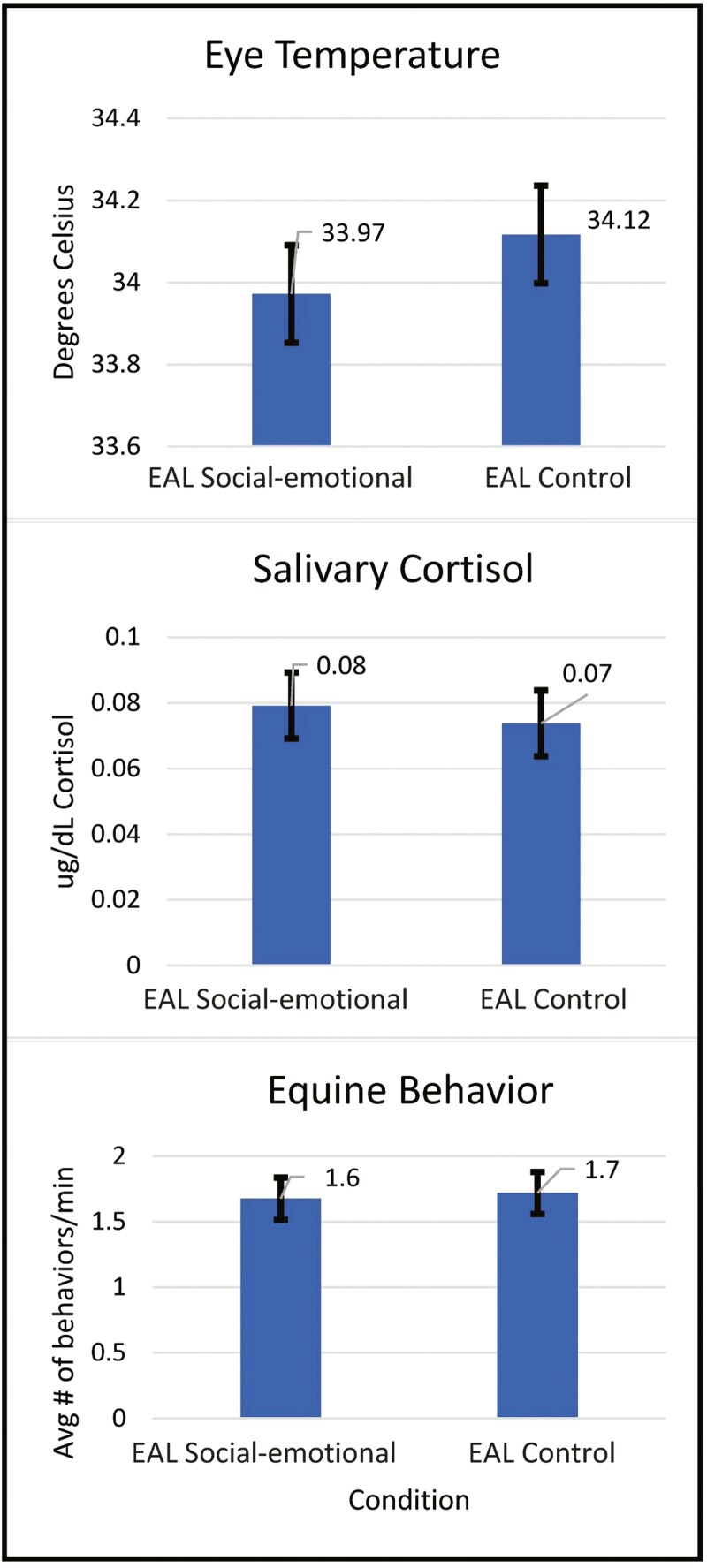
Research question 1: indicators of equine stress during different EAL conditions. Results demonstrate there were no statistically significant differences in eye temperature (p = 0.39), salivary cortisol (p = 0.55), or equine behavior (p = 0.81) during the EAL Social-emotional condition compared to the EAL Control condition.

As illustrated in Figure 2, across all indicators of equine stress (eye temperature, salivary cortisol, behavior), there was no evidence of a main effect for condition, indicating that on average horses did not demonstrate increased stress during EAL Social-emotional compared to EAL Control (eye temperature *F* = 0.73(1,136), *p* = 0.39; cortisol *F* = 0.36(1,85.8), *p* = 0.55; behavior *F* = 0.06(1,135), p = 0.81).

### Research Question 2: Equine Stress During Phases of EAL (Baseline, Tie Rail, Arena Work)

**Figure 3.**
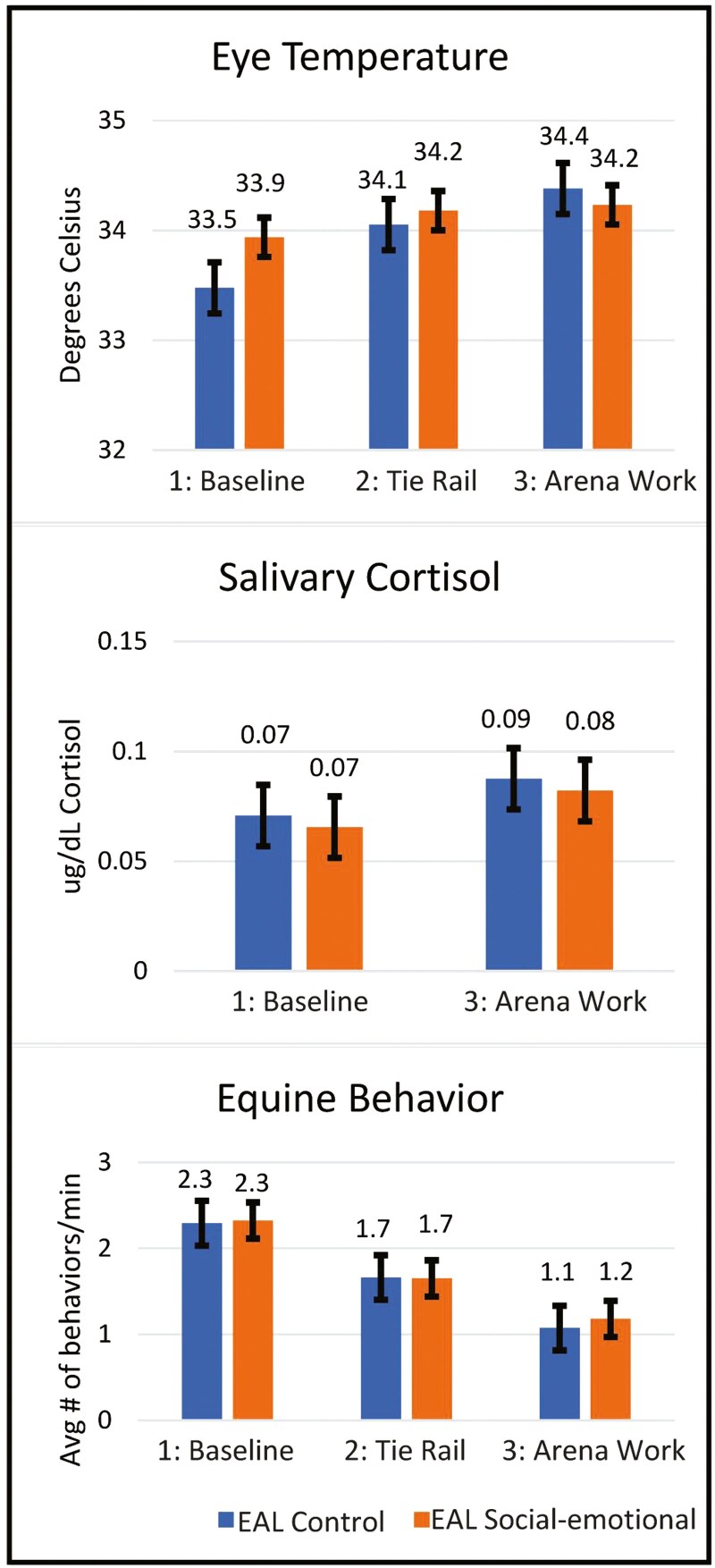
Research question 2: indicators of equine stress during different phases of EAL. There were no significant differences in eye temperature (p = 0.33), salivary cortisol (p > 0.99), or equine behavior (p = 0.96) during EAL Social-emotional compared to EAL Control during three different phases of EAL.

Figure 3 illustrates that across all 3 indicators of equine stress, there were no significant condition by phase interaction effects, indicating horses did not demonstrate increased stress at the tie rail or in the arena during EAL Social-emotional compared to EAL Control (eye temperature *F* = 1.11(2,136), *p* = 0.33; cortisol *F* = 0.00(1,81.7), *p* > 0.99; behavior F = 0.04(2,126), *p* = 0.96).

Although not directly related to our research questions, there were significant phase effects for eye temperature and cortisol (*F* = 4.47 (2,136), *p* = 0.01; *F* = 4.24(1,81.7), *p* = 0.04 respectively). Eye temperature was significantly higher during Phase 3 arena work (M = 34.30°C, SE = 0.15) compared to baseline at the tie rail (M = 33.71°C, SE = 0.14, *t* = −2.91(136), *p* = 0.01). Similarly, cortisol was significantly higher at the end of the session (M = 0.08 ug/dL, SE = 0.01) compared to baseline (M = 0.07 ug/dL, SE = 0.01), likely due to increased physical and mental activity of the session compared to baseline (*t* = −2.06(81.7), *p* = 0.04). While statistically significant, these small increases in eye temperature (0.59°C) and salivary cortisol concentrations (0.01 ug/dL) are well within normal ranges (see Discussion).

Regarding equine behavior, there was also a significant phase effect (F = 16.83(2,126), p=<0.0001). There were significantly more equine behaviors indicative of stress during the baseline phase (M = 2.31, SE = 0.18) than at the tie rail (M = 1.66, SE = 0.18) or during arena work (M = 1.13, SE = 0.18; 1B v 2-TR *t* = 3.18(126), p = 0.01; 1-B v 3-AW *t* = 5.79(126), p=<0.0001). Table 4 provides the percentage of 1-min observation intervals where each specific behavior was observed across experimental conditions and phases. Descriptively, licking and chewing, stepping forward back, head raising, and pawing were more prevalent during baseline phase than at the tie rail or during arena work with EAL participants. There were also significantly more equine behaviors indicative of stress at the tie rail with EAL participants than during arena work (2-TR v 3-AW *t* = 2.54(126), *p* = 0.03). Descriptively, licking and chewing, lip quivers, stepping forward/back, head raises, and neck/body shakes were more prevalent at the tie rail with EAL participants than during arena work (Table 4).

**Table 4.** Percentage of 1-min observation intervals where specific behaviors were observed across EAL phases and experimental conditions

Behavior	Overall	1: Baseline		2: Tie Rail		3:Arena Work	
		EAL Social-emotional	EAL Control	EAL Social-emotional	EAL Control	EAL Social-emotional	EAL Control
Licking and Chewing	45.6%	68.8%	60.0%	35.5%	47.8%	31.2%	30.0%
Lip Quiver	32.7%	35.9%	47.5%	45.2%	34.8%	24.7%	3.3%
Step Forward/Back	27.1%	45.3%	40.0%	25.8%	30.4%	7.5%	13.3%
Head Raise	18.3%	34.4%	10.0%	22.6%	8.7%	16.1%	8.3%
Tail Swish	14.8%	14.1%	15.0%	16.1%	4.3%	17.2%	21.7%
Pawing	6.4%	18.8%	20.0%	0.0%	0.0%	0.0%	0.0%
Head Toss	5.3%	9.4%	0.0%	6.5%	0.0%	7.5%	5.0%
Neck and/or Body Shake	5.0%	3.1%	12.5%	6.5%	4.3%	4.3%	0.0%
Lowering the Head and Neck	2.8%	1.6%	7.5%	3.2%	0.0%	4.3%	0.0%
Itching (Rubbing) Head and Neck	2.7%	0.0%	2.5%	3.2%	4.3%	2.2%	5.0%
Jaw and/or Body Stretch	1.8%	1.6%	10.0%	0.0%	0.0%	1.1%	0.0%
Ears Pinned	0.8%	0.0%	0.0%	0.0%	0.0%	4.3%	0.0%
Kick Threat	0.6%	0.0%	0.0%	3.2%	0.0%	0.0%	0.0%
Bite Threat	0.2%	0.0%	0.0%	0.0%	0.0%	1.1%	0.0%
Head Turn with Ears Pinned	0.0%	0.0%	0.0%	0.0%	0.0%	0.0%	0.0%

### Research Question 3: Equine Stress Indicators Across Time

**Figure 4.**
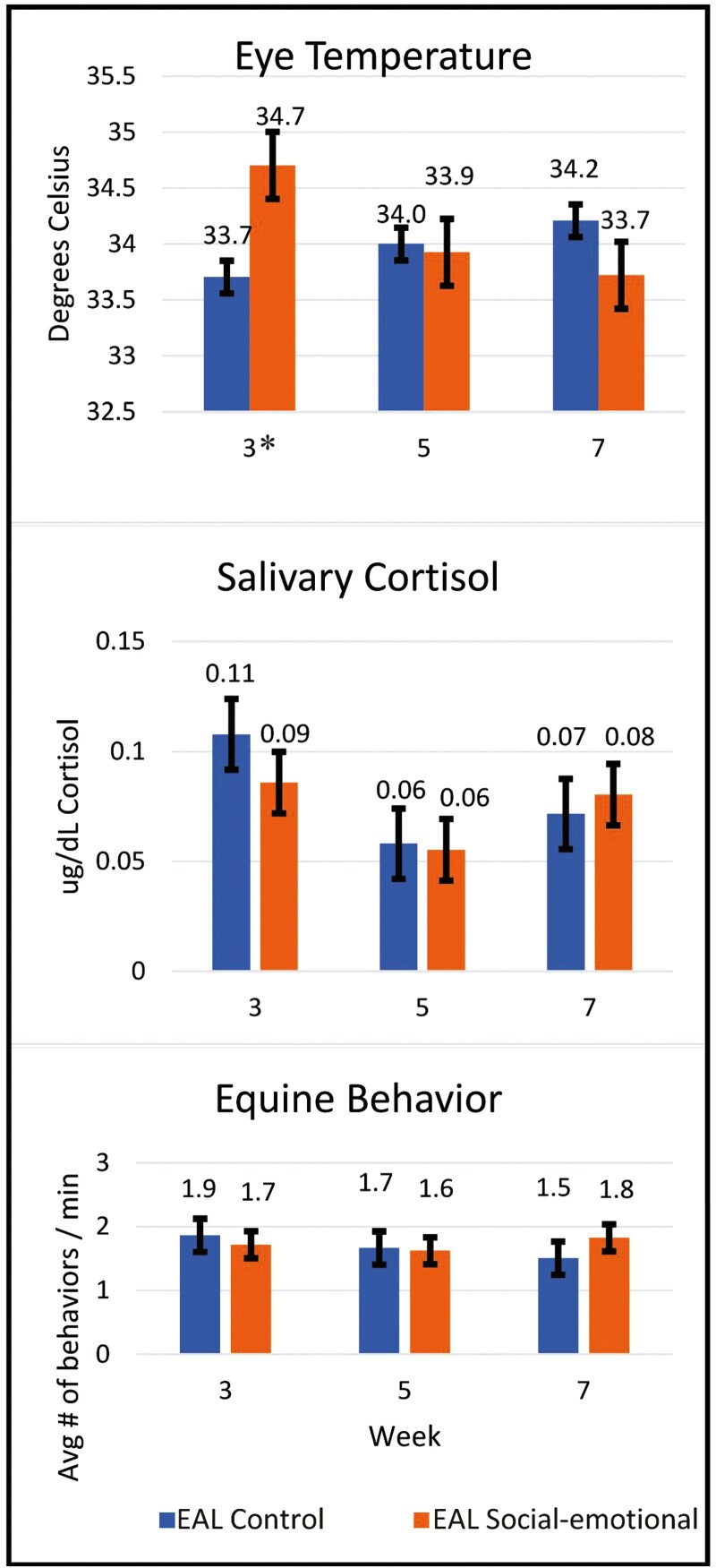
Research question 3: indicators of equine stress across 5 wk of EAL. Results indicate equine eye temperature was significantly greater during EAL Social-emotional than EAL control in Week 3 (p = 0.002). There were no significant differences in salivary cortisol (p = 0.31) or equine behavior (p = 0.5) during EAL Social-emotional compared to EAL control across the 5 wk.

Figure 4 illustrates equine stress indicators across the 8-wk EAL programs. Regarding salivary cortisol and behavior, there were no significant interaction effects of condition by week, indicating that salivary cortisol and equine behavior indicative of stress did not differ by week during the EAL Social-emotional condition compared to the EAL Control condition (cortisol *F* = 1.18(2,81.7), *p* = 0.31; behavior *F* = 0.70(2,127), p = 0.5).

Regarding eye temperature, there was a significant interaction effect of condition × week, such that equine eye temperature was higher in the EAL Social-emotional condition compared to the EAL Control condition in Week 3 (*F* = 6.47 (2,136), *p* = 0.002, *t* = −3.22(136), *p* = 0.002). However, there were no differences in eye temperature between the EAL Social-emotional and EAL Control in weeks 5 and 7 (week 5 *t* = 0.26(136), *p* = 0.79; week 7 *t* = 1.69(136), *p* = 0.09). It is worth noting that Week 3 of the EAL Social-emotional condition was the first day of data collection, and thus the first time that 4 of the 11 horses were exposed to the IRT camera. Eye temperature was slightly higher in these 4 horses (M = 34.91, SD = 0.72) compared to the horses that had been present for the IRT pilot (M = 34.57, SD = 1.16), suggesting exposure to the novel camera may have contributed to this effect.

Although not directly related to our research questions, there was evidence of a significant main effect for week (*F* = 7.77(2,82.20) *p* = 0. 001), such that equine cortisol was significantly higher in week 3 (M = 0.09, SE = 0.01) compared to week 5 (M = 0.05, SE = 0.01, *t* = 3.94(82.4), *p* = 0.001).

## Discussion

This study investigated physiological and behavioral indicators of stress in horses that were integrated into an EAL program for youth with a history of trauma. We examined equine stress across three phases of the program (baseline, tie-rail, and arena work) and collected data across 5 wk (Weeks 3, 5, and 7 of the program). We found no significant differences in equine-stress indicators across any of the three phases during an EAL program for youth with a history of trauma compared to a control condition of EAL for individuals without a history of trauma.

### Equine Stress During EAL Social-Emotional vs EAL Control

Overall, we found that horses did not demonstrate significant differences in stress indicators between the two conditions. Contrary to a previous study by Kaiser et al. (2006) that found increased equine stress during EAS for “at-risk children,” we did not find evidence that horses experienced increased stress while working with youth with a history of trauma and resulting emotional dysregulation, as compared to a different population that commonly participates in EAS. Our findings do seem to be consistent with other literature that found horses did not demonstrate significant differences in salivary cortisol or behavior while interacting in a round pen with individuals with a history of trauma compared to individuals without a history of trauma (Merkies et al., 2018). To understand this apparent discrepancy, the methods of Kaiser et al. (2006) study, which did find evidence of increased stress, merit further consideration. Youth in the Kaiser et al. (2006) experimental group demonstrated detrimental behaviors towards the horses during the sessions, including “subtle signs of impatience, aggressive disposition, or displeasure towards the horse.” There is a growing body of work implicating aversive human emotions (such as anger and/or punitive actions) for negative equine welfare (McLean and Christensen, 2017; Baba et al., 2019; Mellor et al., 2020). It is possible that Kaiser et al.’s (2006) findings were related to negative behaviors expressed by the participants towards the horses and not simply due to youth being “at-risk.” In the current study, anecdotally, participants in the EAL Social-emotional condition demonstrated behaviors indicative of emotional dysregulation, such as frequent irritability, sadness and/or frustration towards the instructor, their peers, or oneself, but generally did not demonstrate negative emotions or aggressive actions towards the horses, which may account for our disparate findings. This underscores the importance of characterizing human characteristics and behavior well in future studies to better understand the effect of different EAS populations on horses, which is in line with scholarly criticism that the term “at-risk” is too vague and requires further definition (Schonert-Reichl, 2000). Regardless of the population, future research and practice in EAS should emphasize the importance of positive human-equine interactions throughout sessions and minimize the risk of aversive behaviors towards the horse.

### Equine Stress During Phases of EAL (Baseline, Tie Rail, Arena Work)

We did not find evidence of increased stress in the horses during any of the three respective phases (baseline, tie-rail, arena work) when working with the youth with a history of trauma compared to the control condition. These are significant findings given that participants worked directly with the horses at the tie-rail, whereas during mounted activities, each horse was directed primarily by the horse leader. We would expect to see an increase of stress at the tie-rail during this one-on-one human-equine interaction if the participants’ previous trauma impacted horse welfare. However, we did not find an increase of stress in the horses when working with youth with a history of trauma during any of the three phases when compared to the control condition.

Although not directly related to our research aims, these data allow for evaluation of stress in the horses during EAL generally, regardless of the type of participants. Interestingly, and perhaps not surprisingly, we found significantly more behavioral indicators of stress during our baseline phase, when horses were alone at the tie-rail, compared to the EAL phases. The most prevalent behaviors included pawing, head raising, stepping forward/backward, and licking/chewing. Consistent with previous findings, where EAS horses demonstrated increased stress (behavior or cortisol) while alone in a round pen or novel stall (Merkies et al., 2018; Rankins et al., 2024), we found that standing alone at the tie rail creates more frustration/behavioral stress than interacting with participants in EAL. This stress is likely related to isolation. Thus, consistent with the Five Domains framework (Mellor et al., 2020), positive human interaction with EAL participants may reduce equine behavioral indicators of stress/tension at the tie-rail.

Furthermore, we found evidence of increased eye temperature during arena activities compared to baseline (alone at the tie-rail) and compared to grooming/tacking at the tie-rail with the participant. Similarly, we found significant increases in cortisol after the session compared to baseline (alone at the tie-rail). Although “normal” cortisol ranges can vary per horse (based on age, health status, and circadian rhythm), previous investigators have reported salivary cortisol concentrations of horses at rest from 0.58 to 3.07 nmol/L (0.021 to 0.111 ug/dL)(Sikorska et al., 2023). The horses in this study had an average concentration of 0.06 ug/dL at baseline and 0.08 ug/dL after the session. Therefore, even at their highest levels, cortisol remained within previously reported ranges of healthy adult resting horses. Moreover, several previous studies have reported higher salivary cortisol levels in horses after exercise (Kędzierski et al., 2013; Peeters et al., 2013); prior research does not make clear if, and how much, low levels of physical activity common in EAS (ridden at the walk and minimal trotting) impact salivary cortisol, but it is possible the slight increases in cortisol could be attributed to the increased physical and mental activity required during the session compared to alone at the tie rail.

Regarding increased eye temperature during arena work, previous studies have reported equine eye temperatures at rest from 29.4 to 37.6°C; our study found a very minor increase from 34.1°C during tie rail activities to 34.3°C during arena work. These are both within the range of eye temperature of resting horses. Previous studies in equine welfare have found eye temperature to increase slightly in response to aversive procedures (Yarnell et al., 2013; Hall et al., 2014), and after training/exercise (Soroko et al., 2016). Again, there is no literature that examines equine eye temperature after very light physical activity common in EAS. Therefore, in the current study it is difficult to determine if the increased eye temperature during arena work can be attributed to increased physical activity, increased mental stress, or both; the current study was not designed to assess this difference (but rather was designed to assess differences due to the human population served). Taken together, our findings of increased salivary cortisol concentrations and eye temperature after arena work deserves further scientific investigation to determine if arena work or interactions at the tie rail impact equine stress differently during EAS, regardless of EAS population served (i.e., individuals with or without histories of trauma).

### Equine Stress Indicators Across Time

Lastly, we found no significant differences in equine salivary cortisol or stress-related behaviors during EAL Social-emotional compared to EAL Control across weeks 3, 5, and 7, nor in eye temperature between weeks 5 and 7, indicating that horses did not experience more stress over time when working with youth with a history of trauma. This contrasts with a recent study that found overall increased heart rate and decreased affiliative behaviors in horses over the course of 10 wk of EAL for “at-risk adolescents,” although this relationship differed by youth’s attachment style (Arrazola and Merkies, 2020). Again, neither Arrazola and Merkies (2020) nor the current study included measurements of youth’s behavior or physiology during the EAL session, important variables to consider in future research which may explain the discrepant findings.

It should be noted that equine eye temperature was higher in week 3 during EAL Social-emotional compared to EAL Control. However, week 3 of EAL Social-emotional was the first day of data collection for all horses, and the first time that 4 of the horses had seen the IRT Camera (as they were not present for pilot data collection day). Given that behavior indicators and salivary cortisol were not significantly different that day, we believe the difference in eye temperature was most likely due to the novelty of the camera and not due to increased stress from participant interactions.

### Limitations and Future Directions

Although we found promising evidence that EAL for youth with a history of trauma does not seem to cause undue stress to horses, our study was not without limitations. First and foremost, the horses in this program were carefully selected for this type of work from a herd of approximately 25 EAS horses. These horses had specific personalities and characteristics that made them more suitable, and perhaps more tolerant, to the emotional state of humans. Although this may have been a limitation in our ability to detect changes in equine stress during EAL for youth with a history of trauma and emotional dysregulation, it also demonstrates the importance of selecting ideal horses for this type of work. Future research in EAS should look at characterizing equine personalities and temperaments to successfully partner horses with humans. Another challenge in our study was our baseline condition. To get the most suitable readings for the thermography camera (i.e., consistent air quality, temperature, humidity, etc.), we chose to gather baseline data at the tie-rail, which resulted in behavioral indicators of frustration/stress. Future studies would benefit from identifying a baseline condition that best reflects a more natural condition for the horses. Additionally, the current study only considered behavioral and physiological indicators of stress; future research should also consider indicators of positive affect in EAS horses. On a final note, although we assessed management conditions, we did not systematically include data that examines the potential presence of management-induced stress, which could have included time budgets, chronic disposition, or cognitive bias. It is possible, and perhaps likely, that stress in EAS horses is more associated with management practices than the services themselves, and thus management-induced stress in EAS horses merits further scientific investigation. The Five Domains of Animal Welfare provides clear guidelines for optimal equine management and has decades of research that illustrates the negative impacts from insufficient management (Mellor et al., 2020).

During observations, researchers (SM and AS) noticed several horses who maintained chronic ear positions during the EAL programs, and thus began recording chronic ear positions within all three phases. Chronic ear position refers to a consistent, prolonged posture of the horse’s ears that may indicate a persistent emotional or physical state. The term “chronic” highlights that the ear position is not temporary but rather sustained over time. Nine of the 11 horses demonstrated chronic airplane ears (ears held sideways; n = 4) or ears slightly back (n = 5) throughout all phases, a condition that seems to be anecdotally evident within the context of EAS and beyond. While ears held sideways has been anecdotally associated with a relaxed state in horses, Fureix et al. (2012) found that lack of ear movement (i.e., chronic ear positions) was associated with a “withdrawn” state in horses, and that such as state was associated with lower plasma cortisol, lower responsivity to their environment, and higher anxiety to challenging stimuli. Thus, chronic ear positions should be further investigated as a potential indicator of a withdrawn state in EAS horses. These findings may be significant to the field of EAS where low-level indicators of stress are difficult to ascertain yet could be contributing to problematic behaviors in horses. While the current study seems to indicate that working with youth with a history of trauma did not contribute to equine stress during EAS, future research can continue to consider other conditions that could negatively impact equine welfare during EAS such as chronic pain (i.e., musculoskeletal and/or gastrointestinal issues) and/or negative learned associations with mounted activities (i.e., aversive training practices and/or improper use of/fit of equipment). Furthermore, the horse leader serves as an additional variable that may be causing stress, whether it be from associations from that specific human-animal interaction and/or conflicting signals from the horse leader and mounted participant; these are important considerations. Future studies in EAS would benefit from investigating these variables, which would provide further evidence and understanding of how various environmental and/or physical conditions, as well as various human interactions (i.e., training methods) can impact the general welfare and well-being of EAS horses.

## CONCLUSION

In the current study, interacting with youth with a history of trauma was not associated with increased stress to horses during an EAL program compared to a similar program for another common EAL population (young adults with developmental disabilities). Moreover, horses integrated into either EAL program did not experience excessive stress, whether at the tie-rail or during mounted work, and regardless of the type of participant (youth with a history of trauma vs adults with developmental disabilities). In fact, all physiological and behavioral indicators observed were within expected ranges for rest and/or low-level physical activity, except when left alone at the tie-rail where we observed increased frustration-related behaviors. Despite this promising data, problem behaviors in EAS horses persist and horse attrition remains a problem. Future research in EAS should explore management protocols and social interactions through the lens of the Five Domains, as welfare of the EAS horse is not only impacted by human-animal interactions during services. To truly understand equine well-being, it is vital we assess all aspects of equine life and do our best to provide for the most precious asset in EAS: the horse.

## References

[CIT0001] Adams, F. 1886. The genuine works of Hippocrates. New York, NY: W. Wood.

[CIT0002] Arrazola, A., and K. Merkies. 2020. Effect of human attachment style on horse behaviour and physiology during equine-assisted activities–A pilot study. Animals 10:1156. doi: https://doi.org/10.3390/ani10071156 32650381 PMC7401529

[CIT0003] Ayala, M. D., A. Carrillo, P. Iniesta, and P. Ferrer. 2021. Pilot study of the influence of equine assisted therapy on physiological and behavioral parameters related to welfare of horses and patients. Animals 11:3527. doi: https://doi.org/10.3390/ani11123527 34944303 PMC8698107

[CIT0004] Baba, C., M. Kawai, and A. Takimoto-Inose. 2019. Are horses (Equus caballus) sensitive to human emotional cues? Animals 9:630. doi: https://doi.org/10.3390/ani9090630 31470656 PMC6770165

[CIT0005] Berfield, J. B., S. Goncharenko, S. R. Forkus, A. A. Contractor, and N. H. Weiss. 2022. The differential relation of trauma types with negative and positive emotion dysregulation. Anxiety Stress Coping 35:425–439. doi: https://doi.org/10.1080/10615806.2021.1964072 34369816 PMC9136915

[CIT0006] Choi, K. R., M. McCreary, J. D. Ford, S. Rahmanian Koushkaki, K. N. Kenan, and B. T. Zima. 2019. Validation of the traumatic events screening inventory for ACEs. Pediatrics 143:e20182546. doi: https://doi.org/10.1542/peds.2018-2546 30837293

[CIT0007] Draaisma, R. 2017. Language signs and calming signals of horses: recognition and application. Boca Raton, FL: CRC Press.

[CIT0008] Frederick, K. E., J. Ivey Hatz, and B. Lanning. 2015. Not just horsing around: The impact of equine-assisted learning on levels of hope and depression in at-risk adolescents. Community Ment. Health J. 51:809–817. doi: https://doi.org/10.1007/s10597-015-9836-x 25698076

[CIT0042] Fureix, C., G. Chapouthier, P. Jego, S. Henry, L. Lansade, and M. Hausberger. 2012. Towards an Ethological Animal Model of Depression? A Study on Horses. PLoS ONE 7(6):e39280. doi: https://doi.org/10.1371/journal.pone.0039280. 22761752 PMC3386251

[CIT0009] Hall, C., R. Kay, and K. Yarnell. 2014. Assessing ridden horse behavior: Professional judgment and physiological measures. J. Vet. Behavior 9:22–29. doi: https://doi.org/10.1016/j.jveb.2013.09.005

[CIT0010] Ho, N. F., J. Zhou, D. S. S. Fung, and P. H. J. Kua. 2017. Equine-assisted learning in youths at-risk for school or social failure. Cogent Education 4:1334430. doi: https://doi.org/10.1080/2331186x.2017.1334430

[CIT0011] Infrared Training Center. 2022. Level 1 Thermography Courses. https://www.infraredtraining.com/en-US/home/training/level-I/2024).

[CIT0012] Kaiser, L., C. R. Heleski, J. Siegford, and K. A. Smith. 2006. Stress-related behaviors among horses used in a therapeutic riding program. J. Am. Vet. Med. Assoc. 228:39–45. doi: https://doi.org/10.2460/javma.228.1.39 16426164

[CIT0013] Kędzierski, W., K. Strzelec, A. Cywińska, and S. Kowalik. 2013. Salivary cortisol concentration in exercised thoroughbred horses. J. Equine Vet. Sci. 33:1106–1109. doi: https://doi.org/10.1016/j.jevs.2013.04.011

[CIT0015] Martin, P., and P. Bateson. 1986. Measuring Behavior, An Introductory Guide. Cambridge, England: Cambridge University Press.

[CIT0016] McDonnell, S. M. 2003. The equid ethogram: a practical field guide to horse behavior. Lexington KY: Eclipse Press.

[CIT0017] McLean, A. N., and J. W. Christensen. 2017. The application of learning theory in horse training. Appl. Anim. Behav. Sci. 190:18–27. doi: https://doi.org/10.1016/j.applanim.2017.02.020

[CIT0018] Mellor, D. J., N. J. Beausoleil, K. E. Littlewood, A. N. McLean, P. D. McGreevy, B. Jones, and C. Wilkins. 2020. The 2020 five domains model: Including human–animal interactions in assessments of animal welfare. Animals 10:1870. doi: https://doi.org/10.3390/ani10101870 33066335 PMC7602120

[CIT0019] Merkies, K., M. J. McKechnie, and E. Zakrajsek. 2018. Behavioural and physiological responses of therapy horses to mentally traumatized humans. Appl. Anim. Behav. Sci. 205:61–67. doi: https://doi.org/10.1016/j.applanim.2018.05.019

[CIT0020] Nobbe, H. 2016. Evaluation of the welfare of the lesson horse used for equine assisted activities and therapies. Murfreesboro, TN: Middle Tennessee State University.

[CIT0022] Peeters, M., C. Closson, J. -F. Beckers, and M. Vandenheede. 2013. Rider and horse salivary cortisol levels during competition and impact on performance. J. Equine Vet. Sci. 33:155–160. doi: https://doi.org/10.1016/j.jevs.2012.05.073

[CIT0023] Perkins, B. L. 2018. A pilot study assessing the effectiveness of equine-assisted learning with adolescents. J. Creativity Mental Health 13:298–305. doi: https://doi.org/10.1080/15401383.2018.1427168

[CIT0041] Pica-Smith, C., and C. Veloria. 2012. " At Risk Means a Minority Kid:" Deconstructing Deficit Discourses in the Study of Risk in Education and Human Services. Pedagogy and the Human Sciences 2:33–48. https://digitalcommons.assumption.edu/cgi/viewcontent.cgi?article=1003&context=hsrs-faculty

[CIT0024] Professional Association of Therapeutic Horsemanship International. 2021. Standards for Certification and Accreditation. 2021 ed. Denver, CO: Professional Association of Therapeutic Horsemanship International.

[CIT0025] Professional Association of Therapeutic Horsemanship International. 2023. Credentialing. [accessed July 10, 2023]. https://pathintl.org/credentialing/.

[CIT0026] Rankins, E. M., K. H. McKeever, and K. Malinowski. 2024. Behavioral and physiological responses of horses to ground-based adaptive horsemanship lessons for veterans with post-traumatic stress disorder (PTSD). J. Equine Vet. Sci. 135:105049. doi: https://doi.org/10.1016/j.jevs.2024.105049 38513814

[CIT0027] Rankins, E. M., C. L. Wickens, K. H. McKeever, and K. Malinowski. 2021. A survey of horse selection, longevity, and retirement in equine-assisted services in the United States. Animals 11:2333. doi: https://doi.org/10.3390/ani11082333 34438791 PMC8388649

[CIT0040] Riele, K.T . 2006. Youth ‘at risk’: Further marginalizing the marginalized?. J. Educ. Policy 21:129–145. doi: https://doi.org/10.1080/02680930500499968

[CIT0028] Roberts, Y. H., M. Ferguson, and C. A. Crusto. 2013. Exposure to traumatic events and health-related quality of life in preschool-aged children. Quality Life Res. 22:2159–2168. doi: https://doi.org/10.1007/s11136-012-0330-4 PMC3616160 23224614

[CIT0031] Schonert-Reichl, K. 2000. Children and youth at risk: some conceptual considerations. In PECERA Symposium Report: Children and Youth at Risk; 2000; Ontario, Canadá. Canadian Education Statistics Council; p. 9–10.

[CIT0032] Seaman, S., H. Davidson, and N. Waran. 2002. How reliable is temperament assessment in the domestic horse (Equus caballus)? Appl. Anim. Behav. Sci. 78:175–191. doi: https://doi.org/10.1016/s0168-1591(02)00095-3

[CIT0033] Sikorska, U., M. Maśko, A. Ciesielska, L. Zdrojkowski, and M. Domino. 2023. Role of Cortisol in Horse’s Welfare and Health. Agriculture 13:2219. doi: https://doi.org/10.3390/agriculture13122219

[CIT0034] Soroko, M., K. Howell, A. Zwyrzykowska, K. Dudek, P. Zielińska, and R. Kupczyński. 2016. Maximum eye temperature in the assessment of training in racehorses: correlations with salivary cortisol concentration, rectal temperature, and heart rate. J. Equine Vet. Sci. 45:39–45. doi: https://doi.org/10.1016/j.jevs.2016.06.005

[CIT0035] Teledyne FLIR. 2024. FLIR Thermal Studio Suite. [accessed April 22, 2024]. https://www.flir.com/products/flir-thermal-studio-suite/?vertical=condition±monitoring&segment=solutions.

[CIT0036] Torcivia, C., and S. McDonnell. 2021. Equine discomfort ethogram. Animals 11:580. doi: https://doi.org/10.3390/ani11020580 33672338 PMC7931104

[CIT0037] Trösch, M., F. Cuzol, C. Parias, L. Calandreau, R. Nowak, and L. Lansade. 2019. Horses categorize human emotions cross-modally based on facial expression and non-verbal vocalizations. Animals 9:862. doi: https://doi.org/10.3390/ani9110862 31653088 PMC6912773

[CIT0038] Wood, W., K. Alm, J. Benjamin, L. Thomas, D. Anderson, L. Pohl, and M. Kane. 2021. Optimal terminology for services in the United States that incorporate horses to benefit people: a consensus document. J. Altern. Complement. Med. 27:88–95. doi: https://doi.org/10.1089/acm.2020.0415 33252244

[CIT0039] Yarnell, K., C. Hall, and E. Billett. 2013. An assessment of the aversive nature of an animal management procedure (clipping) using behavioral and physiological measures. Physiol. Behavior. 118:32–39. doi: https://doi.org/10.1016/j.physbeh.2013.05.013 23685232

